# A Semantic Labeling of the Environment Based on What People Do

**DOI:** 10.3390/s17020260

**Published:** 2017-01-29

**Authors:** Jonathan Crespo, Clara Gómez, Alejandra Hernández, Ramón Barber

**Affiliations:** Department of Systems Engineering and Automation, University Carlos III of Madrid, 28911, Spain; clgomezb@ing.uc3m.es (C.G.); alejhern@ing.uc3m.es (A.H.); rbarber@ing.uc3m.es (R.B.)

**Keywords:** semantic labeling, semantic navigation, mobile robotics, detecting people, environment classification

## Abstract

In this work, a system is developed for semantic labeling of locations based on what people do. This system is useful for semantic navigation of mobile robots. The system differentiates environments according to what people do in them. Background sound, number of people in a room and amount of movement of those people are items to be considered when trying to tell if people are doing different actions. These data are sampled, and it is assumed that people behave differently and perform different actions. A support vector machine is trained with the obtained samples, and therefore, it allows one to identify the room. Finally, the results are discussed and support the hypothesis that the proposed system can help to semantically label a room.

## 1. Introduction

Intelligent robotic systems frequently try to copy human behavior. In the area of mobile robot navigation, this means providing the robot with the ability to understand the surrounding environment in the same way a human does. Semantic navigation [1] deals with this fact.

The navigation system should consist of several modules; one of them is a mapping subsystem [2,3]. Semantic navigation requires the robot to recognize and label places in order to include this information on the map. This semantic interpretation of the environment increases the autonomy of the robot.

The goal of this work is to try to identify different behaviors using the movement of people and background sound, to differentiate rooms. To achieve this goal, the the exact identification of the action that people carry out is not necessary. Thus, the actions are enough for the system to learn. If this ability is attained, the next target is to be able to label places with this information. The proposed system can be helpful in the semantic labeling task. The labeling of places can add a semantic layer to a topological or geometric navigation system; this would increase the efficiency of the navigation system.

**Figure 1:**
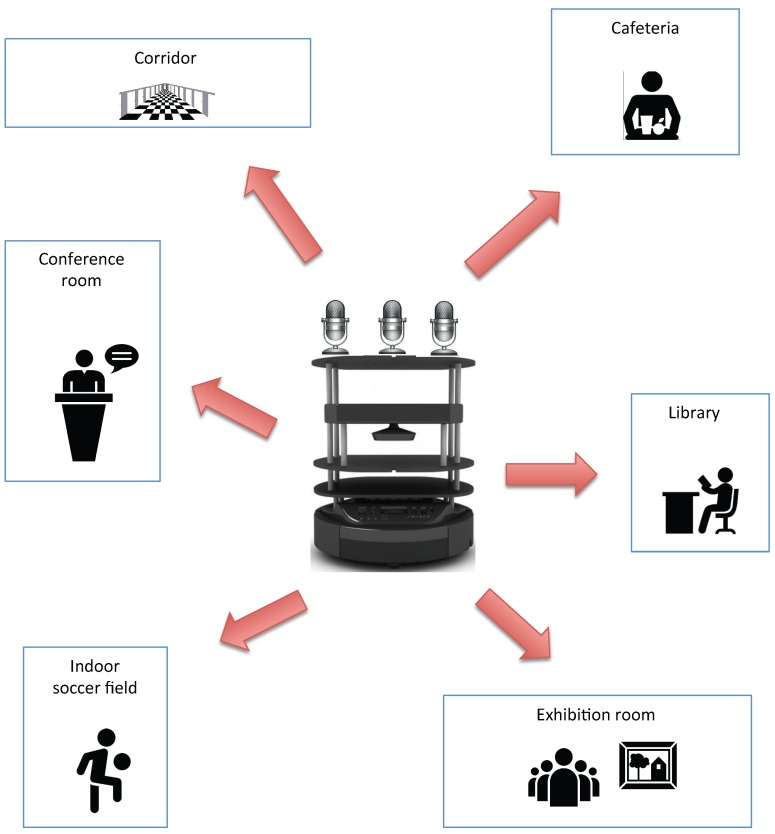
Schematic representation of the classification system of rooms according to what people do.

Other labeling systems are based on detecting elements within the environment. For example, object detection systems provide much information to the labeling task because each type of room usually contains specific objects (the kitchen contains cooking utensils, the living room a television, etc.) [4]. However, in this paper, a new approach is proposed. This approach is based on analyzing what people do. Then, it deduces what the type of environment could be. These features are represented in Figure 1. This is a new point of view, because until now, the semantic labeling of places depended only on the objects contained in that location and on its physical characteristics. In semantic navigation, the trend has been to evaluate the labeling task based on the number of features or information about the environment that the system is able to handle. These features are typically [5] the place appearance, the place geometry, the object information, the topology, the human input, the segmentation, the conceptual map, the uncertain objects, the inferring properties, the concepts acquired, etc. The more features or approaches for the system to handle, the more information the semantic map has to create. The approach of the system described in this paper adds a new feature to the one listed above, that is the information from people acting in the environment. This feature had not been previously taken into account. This also provides great dynamism in the labeling task. For example, a place may be labeled as a cafeteria, because it detects a busy place; a crowd of people moving to and from the bar. If that same place suddenly ceases to be crowded and noise decreases, it can be labeled as a reading area. If there is enough silence to read in one place, it may well be a place for reading. Anyway, this system is a good support for the semantic labeling.

The proposed system has been tested in six different types of environment: cafeteria, library, corridor, exhibition room, conference room and an indoor soccer field. One of this new system’s goals is to ensure that the robot can differentiate each environment.

### Related Works

In recent decades, researchers focused on cognitive navigation, an area that combines the movement of the robot with a high level of environmental perception capability. One of the reasons that led these researchers to depend on the ability of perception is the labeling and classification of places. In [6], the authors faced the problem of cognitive or semantic navigation by decomposing it into discrete tasks. In that paper, the goals of the recognition of places and place categorization are discussed. The navigator requires using robust and competent machine learning techniques to deal with any dynamic change of the explored environments, and therefore, the robot should be able to categorize and label places.

The literature on place labeling methods for robot navigation is extensive [7,8,9]. A trend is to identify regions of interest in the environment, such as floor, walls and doors [10]. However, although this gives the system some knowledge concerning navigation, it does not categorize the place. This task is approached in [11], where they distinguish corridors, office rooms, lecture rooms and doorways. In addition, the authors weigh the advantages and disadvantages of using different sensors for semantic labeling of places. For example, the works described in [12,13] are based on vision sensors, and the works described in [14,15] are based on laser range finder data. In addition, they discussed a labeling system based on a multi-sensory approach in [16].

The labeling of places is an objective widely studied. In [17], the Hough transform is used to identify corridors. In [18], a neural network is trained with odometry information to detect the position of the robot. One of the fields benefiting from semantic labeling is mobile robot navigation, making it an area of great interest. Topological navigation systems can be built from the results obtained from nodes labeled with the proposed method in this paper. Semantic navigators can use the method presented in this work, and it can be built on a topological or geometric navigator. In [14], data from a 360° planar system are used to distinguish between room, corridor, hallway door and places. To achieve this, only geometric data are used. Other works describe how to adequately incorporate depth information into the local model, pairwise and order interactions. In [19], a model is proposed following this line. It improves scene labeling techniques. RGB-D cameras are used in more works, trying to develop or improve mapping techniques. This is the case of [20], where a complete 3D mapping system is presented. This system combines visual features and shape-based alignment. In this paper, it is considered that every labeling systems is limited by the use of a few sensory information sources and types. Additionally, if more sources of the data environment are obtained, this would improve the labeling abilities. Other ways of semantic labeling focus on object recognition, as in [21]. The Haar features are used for the number of specific objects in the environment. Adding the type of information managed by the system described in this paper, labeling of places can be even more refined.

None of the systems described above have included techniques designed to find patterns in the actions of individuals, in order to label a room based on what people do there. Our system aims to open a way toward this direction.

Detection of people is a widely discussed issue, but it has not specifically been used for semantic labeling. A complete system can be found in [22]. Blob segmentation, head-shoulder detection and a temporary refinement is carried out. However, in this paper for the first approach, a person detection algorithm is chosen. This algorithm is only used to detect legs. Other authors have chosen the same option [23].

Regarding the utility of the background noise information, previous works of mobile robotic systems involving microphones have mainly been focused on sound localization and human-robot interaction by speech. Regarding sound localization [24], using several distributed microphones allows one to derive the position of an emitting acoustic source in a given environment, as shown in the works developed in [25,26,27]. This principle has successfully been used in fields, such as underwater sonar, teleconferencing or hearing aids, because it can be used to detect multiple active and passive sources. Regarding human-robot interaction, the importance of a symbiosis between humans and robots leads to an improvement of the perceptual capabilities of robots. In particular, hearing abilities are being studied so that interaction is possible in real-world environments [28,29,30].

Learning categories and subsequent real-time labeling are solved by a SVM (Support Vector Machine). The SVM is similar to the methodology used in the works of other authors [15]. When semantically classifying the environment, a problem is encountered. This problem is solved using range finder data in wheeled mobile robots. An SVM classifier in a supervised way to minimize the classification error is trained. The raw data are transformed into a group of simple geometrical features from which the classification of places could be extracted. These features are named as simple because they are single-valued. Finally, a classifier between different rooms and the corridor is obtained. The data from which their SVM is trained are based on the area, perimeter, compactness, eccentricity and circularity (defined as $\frac{perimeter^{2}}{area}$) extracted from the places.

However, the possibility of using other learning tools should not be underestimated. There are other works more focused on the recognition of patterns with neural networks for the classification of scenes. In [31], more than seven million labeled scene images are used. Deep convolutional neural networks for scene recognition and deep features are used. One of the goals of [31] is to demonstrate that an object-centric network and a scenic-centric network learns different features. For these results, the features extracted from the pre-trained network and a linear SVM are used. Although the author does not consider the main objective of this paper, the techniques used are interesting. The characteristics used in this paper are of a different nature from those of other systems that can be found in the state of the art. Another way of labeling of places that uses convolutional neural networks is found in [32]. However, in this case, a new learning feature called spatial layout and scale-invariant convolutional activations is presented. This incorporates an interesting spatially unstructured layer to introduce robustness against spatial layout deformations.

## 2. Materials and Methods

The system presented in this paper labels the environment in terms of what people do in it. What people are doing is distinguished according to the background noise, the number of people and the movement of these people. Different activities provide different sensory data. This allows the system to perceive that the actions performed in those places are different. Therefore, they are places with different functions. Therefore, the place is labeled according to the actions people carry out in them.

### 2.1. Complete System

The complete semantic labeling system consists of several modules. The idea is that the system can deduce the type of room, according to the activity people are performing in the room. Finding out what a group of people is exactly doing in a room can be hard. However, the robot can affordably deduce that people perform different actions. Although, the robot does not know what exactly those people are doing. To achieve this, this paper focuses on the number of people, how many meters these people have displaced and background noise. If the system focuses only on that, it can know that in certain rooms, people are carrying out different actions. Thus, these rooms are labeled.

**Figure 2:**
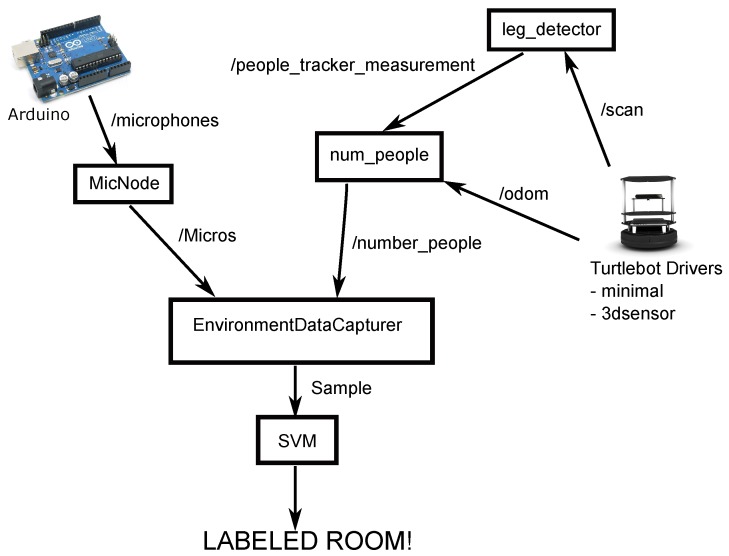
Complete system diagram.

The modules and elements of the complete system are shown in Figure 2. They are:

- A mobile robotic platform: A Turtlebot-2 with the collection of software frameworks known as Robot Operating System (ROS) is used. It has the drivers *minimal* and *3dsensor* operating.
- A people detecting node: The *leg_detector* node (see Section 2.2) has been chosen for this paper. It has been obtained from the LIDAR web. It is open software.
- The *num_people* node: It is responsible for obtaining the information of the detected people and their movements in a given time interval. The sampling is performed when the robot is stationary to avoid the displacement of the robot to alter the sample. Another option may be to consider the movement of the robot to cancel it, but this idea has been rejected because it was not considered necessary and would increase the run time.
- Set of microphones and Arduino: In Figure 2, Arduino is shown. A structure with three microphones that are attached to Turtlebot has been designed to sample the background noise. The Arduino transmits the data obtained from microphones by a message on the topic/microphones.
- The *MicNode* node: This node samples the information received from the microphones and sends each sample in a message on the topic/Micros.
- EnvironmentDataCapturer node: This node receives data samples of noise and movement of people and handles and merges both data in a synchronous sample. This sample is stored in a file to train the support vector machine or it can be sent to a trained SVM to classify a room.
- SVM node: This is the module that manages the support vector machine.

The system takes samples when the robot is motionless. The robot can be teleoperated around a room and stopped at some positions to obtain the samples. The robot can also be controlled by a modified wandering node occasionally to stop the robot and orientate toward the wider visual area.

### 2.2. People Detection

**Figure 3:**
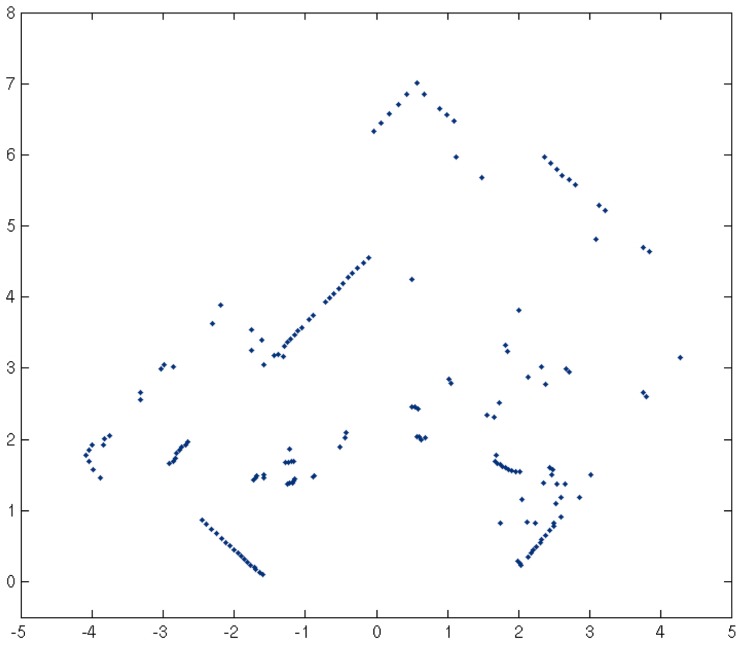
Example scan from a typical office with people in [34].

Since this semantic labeling system needs to identify what the people are doing, the first issue is to include a people detection system. A method that is available at the official ROS website (http://wiki.ros.org/leg_detector) is used. This algorithm is based on leg detection to infer that a person has been perceived. The administrator briefly commented on this method in [33]. These authors needed a people detection system, as well, and reduced the problem to that of detecting legs. Their leg detection technique is based on the algorithm of Arras et al, [34] and extends an implementation developed at Willow Garage by Caroline Pantofaru. A group of low-level classifiers to estimate the probability that the laser scan data obtained are reading a leg or not is all that is used. The next step is to analyze these leg probabilities, focusing on distance constraints. An algorithm pairs the individual legs that correspond to a person (under their assumptions) and tracks the resulting leg-pairs. Thus, the legs detector algorithm identifies in which position people are in a room as in Figure 3, using only laser scan information.

The node *num_people* is subscribed to *people_tracking_measurements* and *odom* topics, as shown in Figure 2. The purpose of this node is to publish the number of people in a room at a certain moment. It also publishes data about the amount of motion of those people. It receives information from the node *leg_detector*, an array with all detected persons. This array contains the identifier information of each person, its reliability and its current position. It also receives information from the *odom* topic to get the current position and velocity of the robot.

**Figure 4:**
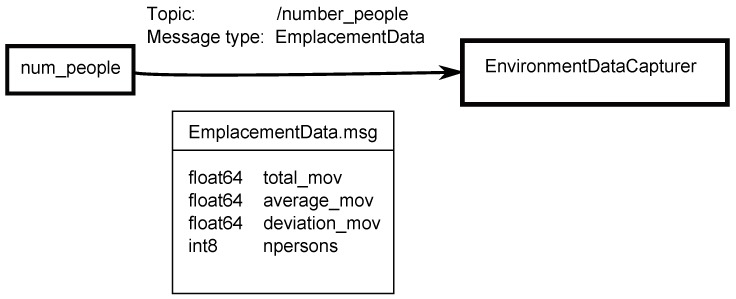
EmplacementData message published on/number_people topic.

The node works by collecting all of the data received during a predefined time interval *sample_time*, as long as the robot is motionless. During this interval, an array formed by DetectedPerson objects, with all of the people detected is stored. New data from topic *people_tracking_measurements* are published by the *leg_detector* node in the form of a PositionMeasurementArray message. This message contains an array with the information of every detected person and his/her current position. Each person is differentiated by an identification (id). The node checks whether the detected persons at the moment were included in the array. The positions of the people who have a recognized identifier are updated, and new people are added. When the interval concludes, the amount of the motion of detected people is estimated in that interval. Then, the message to publish in /number_people topic is prepared. This message is the EmplacementData type (Figure 4), and it contains information of the total amount of movement of all of the people, the average person motion and typical deviation. Obviously, it also has the information of the total number of detected people in the interval.

The displacement detected by num_people node takes into account the movement in the two dimensions of the ground. A person $p_{i}$ is detected in a time interval *t*, and the person has moved a certain distance $dp_{i}$, as represented in Equation (1).

(1) $$\forall p_{i},dp_{i}=\sum_{t=0}^{MAX\_TIME}|PX_{new}-PX_{old}|+|PY_{new}-PY_{old}|$$

The movement of a person in a sample is the difference in absolute value between the current position in the *X* axis ($PX_{new}$) and the previous position ($PX_{old}$) plus the difference in absolute value between the current position on the *Y* axis ($PY_{new}$) and the previous position ($PY_{old}$).

(2) $$D_{t}=\sum_{i=1}^{Np}dp_{i}$$

Samples are taken at a configurable time interval. The experiments conducted were performed with a 3.5-s interval. The total displacement $D_{t}$ is the sum of the displacements of all identified persons Np in that time interval in Equation (2).

(3) $$\bar{x}=\frac{D_{t}}{Np}$$

The calculation of the arithmetic mean is performed to obtain the average displacement of each person in the sample (Equation (3)), and the standard deviation (Equation (4)) is added to provide more information to the sample.

(4) $$\sigma =\frac{\sum_{i=1}^{Np}{\left(dp_{i}-\bar{x}\right)}^{2}}{\bar{x}}$$

The node *num_people* also allows one to configure the coefficient of certainty to identify a person. In the experiments conducted, the coefficient is adjusted to 70%. The implemented person counter program subscribes to a topic that publishes the people detection node, which contains a field that indicates the degree of certainty about that detection. The counter program has been created with an adjustable parameter to modify the minimum degree of certainty that is accepted in order to consider a detection as positive.

### 2.3. Background Noise Acquisition

**Figure 5:**
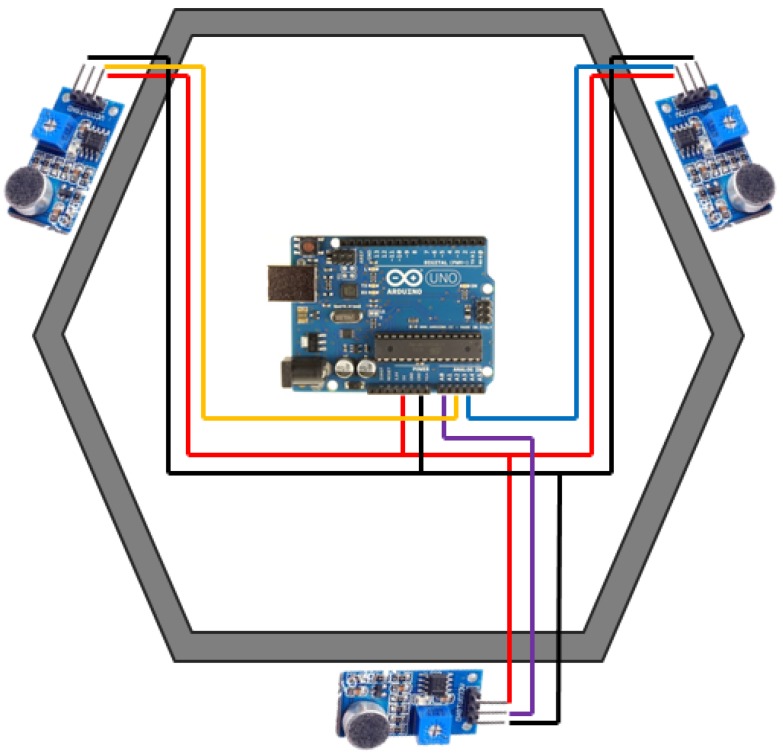
Schematic diagram of the microphones’ structure.

For this paper, a microphone array has been developed. The whole noise reception device is formed by three microphones and an Arduino UNO board to process the data acquired from the microphones (Figure 5). This device is designed as a 3D-printed ring to be placed on top of Turtlebot robot, and noise is registered when the robot is motionless. The NodoMic node is transmitting noise data continuously with a predefined time interval, but the node that takes background noise and people samples saves the data when the odom topic indicates a current velocity of zero meters per second. This procedure avoids interferences by robot displacement noises, reducing the alterations of sound data and of the movement of people. Movement of people is easier to calculate since the robot is not moving, and it acts as a fixed reference point. The three microphones are mounted in the 3D-printed ring, and they are oriented in different directions, so the position of the sound source can be easily estimated. The purpose of the sound reception device is to capture background noise and estimate the position of noise sources from the difference in the intensity captured by each microphone. From these two concepts, the system is able to learn about the acoustic situation of the environment, without the need for complex source location algorithms.

The NodoMic node receives information from the topic *microphones*, which is sent by the Arduino that is connected to the microphones. Microphones sample the background noise for a predefined time interval. The node processes the information and sends it in the topic *Micros*.

### 2.4. Information Multimodal Fusion

The environmentDataCapturer node is responsible for gathering all of the information about the environment and managing samples to label the room. Therefore, this node is subscribed to all of the topics that can provide information about what people are doing. It receives information from *number_people* and *Micros* topics. The first step to obtain a reliable sample is to ensure that all of the information is concurrent. The reception of messages on topics is asynchronous; this implies that these data receptions must be managed. A constant *max_time* for the range of time is defined. If the data received from the two topics (noise and people data) reach the node with a difference of less than this time range, then the data are considered concurrent. In addition, a constant sampling time *time_between_samples* has been set to define how long the system waits before taking a new sample.

This node achieves the fusion of the data received and obtains the samples. First, these samples are collected in a file, which will be accessed by the Support Vector Machine (SVM) programmed for the training process. Once the SVM has been trained, samples can be classified, and rooms are labeled. This process can be performed offline, testing with the samples’ file.

The structure of the features vector stored for each sample is shown in Equation (5). $M_{1}$ is the microphone1datum; $M_{2}$ is the microphone-2 datum; $M_{3}$ is the microphone-3 datum; $N_{p}s$ is the number of people in the sample; $D_{t}$ is the total displacement of the people; $\bar{x}$ is the arithmetic mean of the displacement; and *σ* is the standard deviation.

(5) $$Features\_vector=\{M_{1},M_{2},M_{3},N_{p}s,D_{t},\bar{x},\sigma \}$$

### 2.5. SVM Training

A program implementing a support vector machine has been developed. The SVM is obtained from the open source library of programming functions mainly aimed at real-time computer vision called OpenCV. The SVM implementation offered by this library has been widely used in other works (especially in computer vision) [35,36].

SVM parameters have been established as the library used allows one to configure them. These parameters are:

- svm_type: The is the type of SVM formulation. The set value is CvSVM::C_SVC. This choice is for n-class classification, and it allows imperfect separation of classes with a penalty multiplier for outliers.
- kernel_type: This is the type of SVM kernel. The chosen value is CvSVM::LINEAR. This configuration is the fastest option. No mapping is carried out; linear discrimination is done in the original feature space.
- term_crit: This is the termination criteria of the iterative SVM training procedure which solves a partial case of the constrained quadratic optimization problem. The tolerance and the maximum number of iterations are also set. In this work, the type of termination criteria is CV_TERMCRIT_ITER; this means that the algorithm always ends after some number of iterations. Seven thousand iterations are considered the maximum set.

Each training sample for the SVM algorithm is made up of one observation $D_{i}$ and its classification $C_{i}$. The set of training examples is then given by Equation (6) where Υ is the set of classes. In this work, it is supposed that the classes of the samples for training are known a priori. The goal is to learn a classification system that is able to generalize from these training examples and that can later classify unseen places in this environment or other environments.

(6) $$S=\{\left(D_{i},C_{i}\right):C_{i}\in \Upsilon =\left\{Library,Cafeteria,...\right\}\}$$

When the program is run, a sample file name must be typed at the command prompt. The sample file introduced is generated by the environmentDataCapturer node. In the offline execution (used in the experiments), the program also requests the percentage of samples that will be used to train the SVM. The remaining samples make up the test set. Each sample has a probability of belonging to one of the sets that is determined by the percentage entered. This allows running the same file several times to obtain different results.

**Figure 6:**
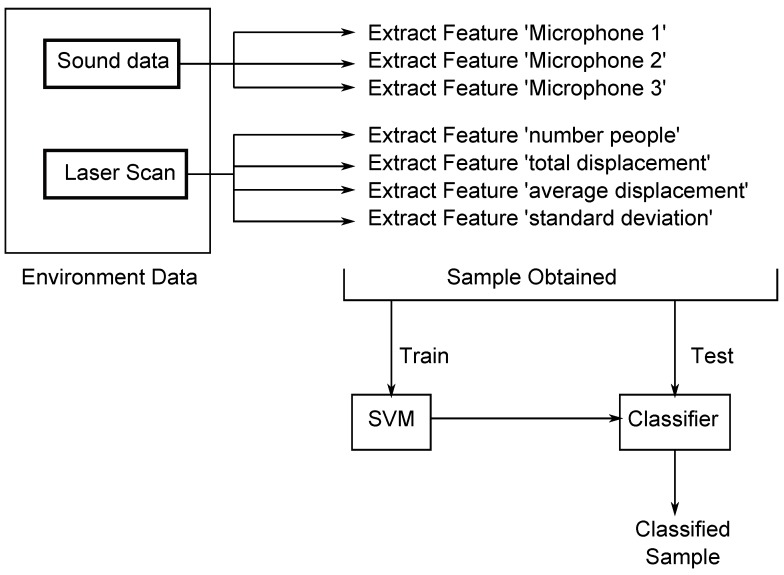
Generation, training and classification of samples.

The file received has been constructed from the obtained samples. These samples of the environment are perceived by the sensors of the robot, and the SVM is trained with them. When the training stage is over, a sample can be classified. This process is illustrated in Figure 6. The file that the SVM receives has the following format for each record:

- Room ID: The class of the room where samples are being taken is known. This learning is supervised. The first element of the file is the known room classification.
- Microphone-1 datum: The sound system consists of three microphones. Microphone-1 is located in the front of the robot.
- Microphone-2 datum: This datum corresponds to the microphone that is located on the right side.
- Microphone-3 datum: This is the datum from the microphone located on the left side.
- Number of people: This is the amount of people detected in this sample.
- Total displacement: This datum is the sum of the displacement of all people in the sample. Therefore, a measurement of the movement recorded in the room is obtained.
- Average displacement: This is the arithmetic mean, the total displacement divided by the number of people. It is an estimation of the average movement.
- Standard deviation: This is the standard deviation of the amount of displacement of all of the people in the sample.

## 3. Results

### 3.1. Basic Test Description

In the first approach, three environments were tested. The amount of displacement of each detected person and the background noise were measured and recorded. The number of detected people, the average and total displacement of these people and the standard deviation of the data were also simultaneously registered.

**Figure 7:**
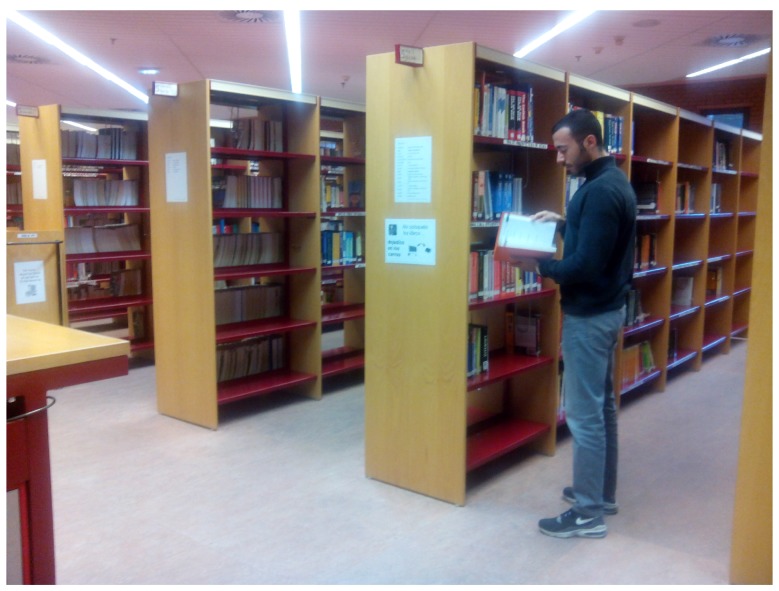
Obtaining library samples.

**Figure 8:**
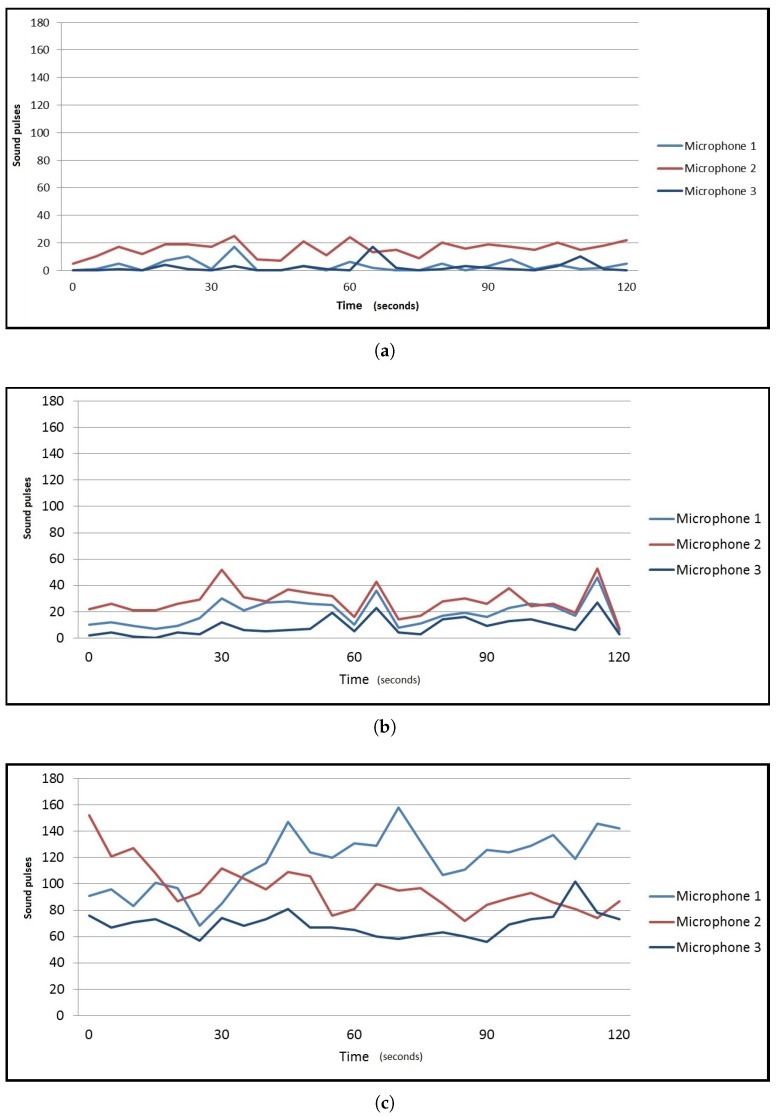
Background noise samples. (**a**) Library noise samples; (**b**) Corridor noise samples; (**c**) Cafeteria noise samples.

Regarding the library environment, one hundred and one samples were taken (Figure 7). Background noise data are shown in Figure 8a, showing that it is a quiet environment. This scenery is chosen because a quiet environment could modify people behavior. The data were obtained by placing sensors in different parts of the library, so as not to disturb the students who were in the library.

**Figure 9:**
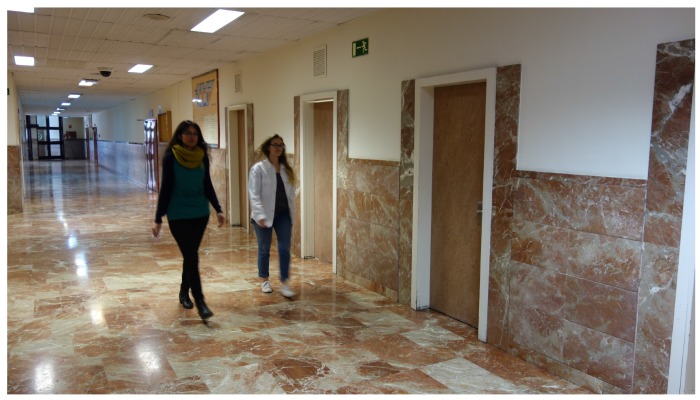
Obtaining corridor samples.

**Figure 10:**
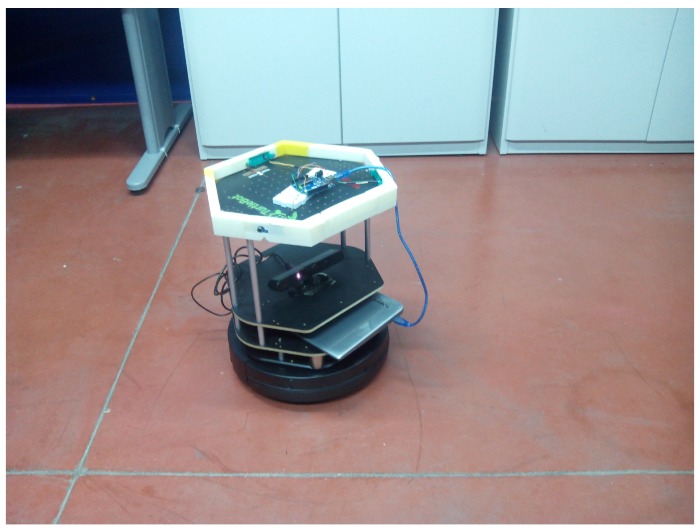
Turtlebot equipped with microphones and Asus 3D-sensor.

**Figure 11:**
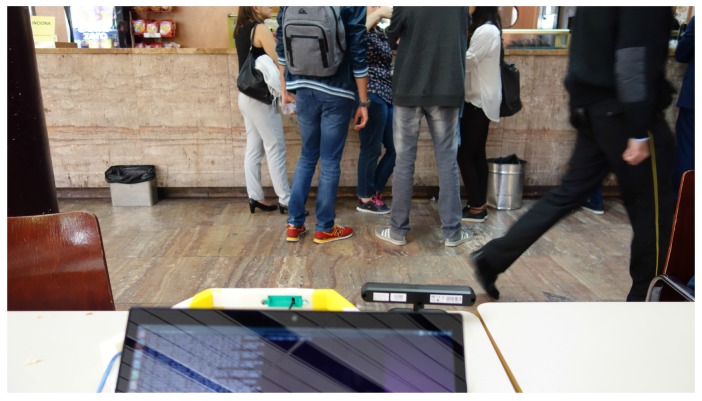
Obtaining cafeteria samples.

In the corridor environment (Figure 9), one hundred samples were taken. Figure 8b shows the background noise data obtained. The same amount of samples was processed in the cafeteria environment. Results are shown in Figure 8c. To obtain the data in the cafeteria, the robot fully equipped with the sensory system (see Figure 10) was teleoperated to reach different zones of the environment where the robot took a certain amount of samples. Some sensors were manually placed (Figure 11). In the corridor, the sensory system was placed at a specific point. The cafeteria is considered a potentially noisy environment, and the background noise in the corridor varies, but the displacement of people is supposed to be greater.

**Figure 12:**
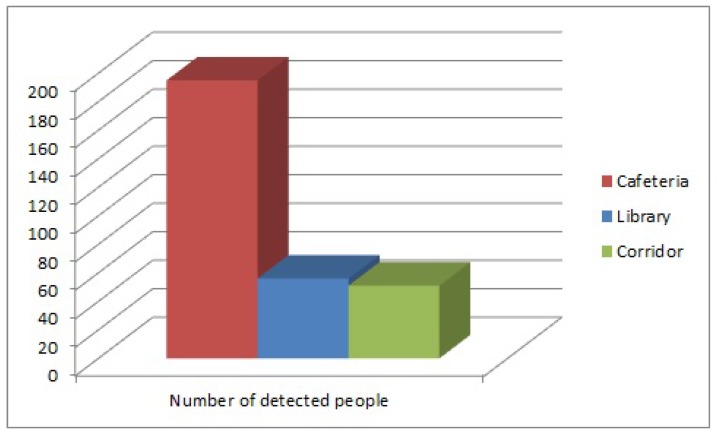
Detected people.

**Figure 13:**
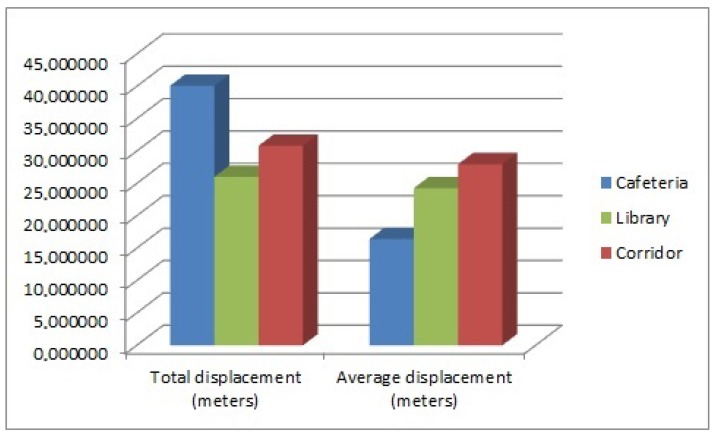
Displacement samples.

**Figure 14:**
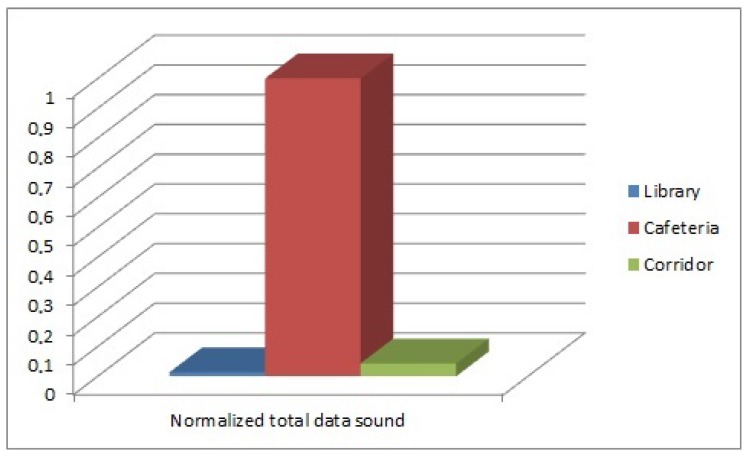
Sound data of the three environments.

The number of detected people in each environment is shown in Figure 12. The data related to the detected displacement of people in each environment are shown in Figure 13. The background noise data obtained from the three microphones of every sample were added and divided by the number of samples. The result of the sum is normalized and shown in Figure 14. While at the library and the corridor, the level of background noise was similar, an important difference in the cafeteria is observed. Therefore, the cafeteria environment can be labeled only using the background noise data. The inclusion of people observation improves the task of labeling, as discussed in the Section 3.2.

### 3.2. Advanced Test Description

After the first test was accomplished, new experiments were conducted to clarify some aspects of the semantic labeling. For example, the question about the combination of the features of the environment, such as the background noise and movement of people, actually improved the effectiveness of the classification task. The improvement is because samples were obtained from two environments. In these two environments, the background noise and the movement characteristics of people are apparently different to classify the environments. The SVM was tested with a set of experiments for an ablation study. The first experiment was conducted without background noise data; then, in another set of experiments, without people data and then the last experiment with all of the data.

**Figure 15:**
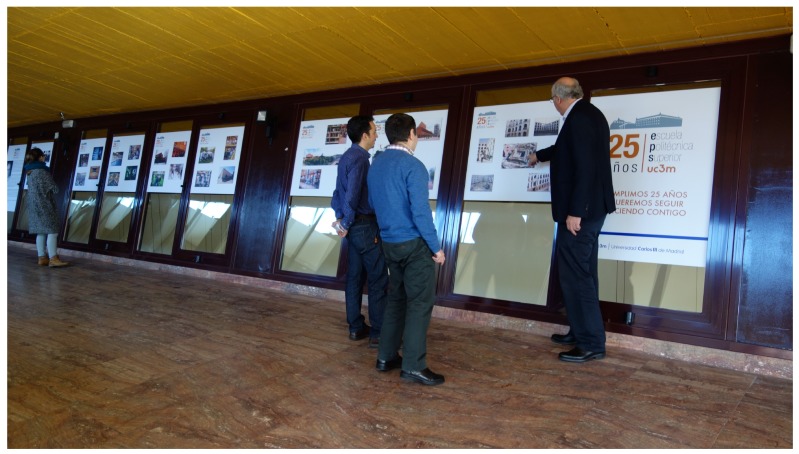
Exhibition room.

These two environments were the corridor and a new environment labeled as exhibition room (see Figure 15). At the time of testing, the SVM was trained randomly in 70%, and 30% was left saved for the experiment set.

More environments (exhibition room, indoor soccer field and conference room) were included to test the effectiveness of the system to classify more environments. A new set of experiments was also conducted.

**Figure 16:**
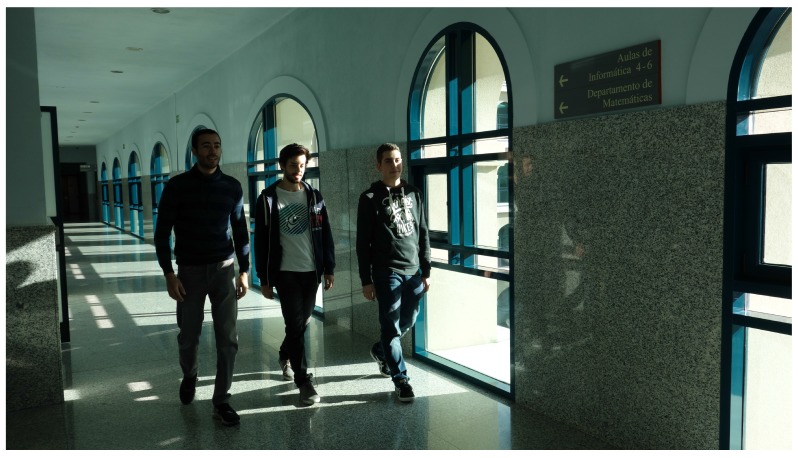
New corridor.

Another unexplored aspect of the previous experiments’ section is the ability to identify places whose samples obtained were not used to train the SVM. The SVM was trained with samples obtained in a different place at a different time. To check this, corridor samples were taken at a new location, shown in Figure 16. A test was run training an SVM with all of the samples of the corridor obtained from the basic experiments. To check if the trained SVM, with all of the samples, was able to identify the new corridor, samples of the cafeteria were added.

### 3.3. Results’ Discussion

The test results are presented in a confusion matrix to assess the validity of semantic labeling based on background noise information and based on the displacement of the detected people for each particular case. Classifiers were created by SVMs, recording all samples taken in a room type. Later, the classifier has been trained with 70% of these samples, randomly selected. The remaining 30% of these samples has been saved to test the classifier. The process was repeated twelve times for each environment, so twelve classifiers were obtained for each case. Thus, the results have been studied avoiding the bias that a single classifier could have generated.

- Library vs. cafeteria:
  The first aim is to ensure that a trained robotic system can differentiate room types with the sensory information available. It is considered that the interpretation of the information is focused on deducing what people are doing in the room. Deducing what people are exactly doing is difficult, but realizing that what people do is different in one place and another is easier. The first test was designed to check that very different places were well labeled. The chosen places were the library and the cafeteria. They are places where people do different things. This is observed in what the sensory system perceived: noise level and the amount of people displacement. Intuitively, a cafeteria is louder, and there are more people per square meter. In a library environment, sound is lower; usually, there is more space between persons, and people move less. Even if a person crosses in front of the sensor, it is estimated that his/her speed will be lower. The results about differentiating these two types of places is reflected in Table 1, which shows that 96.8% of samples were correctly classified. Therefore, 97.22% of library samples and 96.29% of cafeteria samples were well classified. This table shows the results of one of the twelve classifiers generated. Table 2 shows the sum of all of the results of the twelve classifiers. Although there are classifiers better than others, all of them offered very good results. The success rate in this experiment was 98.85% of rooms correctly identified. It is a very good result, but in this case, the classifier should work very well because it was the more intuitive case.
- Library vs. corridor:
  The second aim is to provide a difficult case to the system. The corridor can also be very quiet, and samples were taken at a time of low traffic. In addiction, there are people who just walked in the library in front of the sensor, which is the same action performed in the corridor. The last element that adds difficulty to this environment is identifying seated people in the library at some distance. This is difficult for the system due to sensory limitations. The Asus sensor is designed to work in a range of only three meters, and the people detection algorithm is focused on leg detection. Therefore, it is considered a challenging test. The result, however, is better than expected. Some of the twelve classifiers generated good results. One of them is shown in Table 3. Considering several good classifiers, the results are similar. The rate of success is shown in Table 4; if good classifiers were chosen, the rate is 91.5%. This is a high value; only 13.2% of corridor samples were classified as library. The sum of all classifiers, both good and bad ones, offers the result shown in Table 5. The overall success rate, including the worst classifiers obtained, is 86.9%.
- Library vs. cafeteria vs. corridor:
  The next aim is to check the effectiveness of differentiating several types of room at the same time. Simple and complicated cases have been combined to differentiate library, cafeteria and corridor environments. Choosing a good classifier among the twelve classifiers generated, the results are shown in Table 6. If several good classifiers are combined, the results are shown in Table 7. The sum of all results of the classifiers generated is shown in Table 8, which shows a success rate for room classification of 91.6%.

#### Advanced Test Results

As seen in Section 3.2, another set of tests has been carried out to check some details of the system operation.

- Ablation study: The environments chosen for this test are a corridor and the exhibition room. Table 9 shows the result of the classification tests when the background noise data are removed. The classification ratio is low, but not bad. Table 10 displays the result of the classification when data relating to people and their movement are removed. The ratio is worse than in the previous case, especially trying to classify the exhibition room. In any case, when all of the data are combined, the ratio rises considerably, as shown in Table 11.
- Tests with more environments: A battery of tests has been conducted generating 10 classifiers from the samples taken in the environments exhibition room, indoor soccer field, conference room, library, cafeteria and corridor. Table 12 collects the sum of the 10 classifiers generated. Tests on a different environment from the training set: An SVM has been trained with all samples from the cafeteria and from the corridor of the basic tests. In this experiment, only one classifier can be generated, since by taking 100% of samples considered from both training environments, there are no random combinations. The test was performed with 100% of the samples taken in the second corridor; in total, 93 corridor samples for the test. The result is shown in Table 13.

The advanced tests allow one to verify that there are environments easily identifiable with the proposed system and that the fusion of the variables considered in this paper can improve the identification that could be made with the variables separately. In addition, the identification of a room where samples have not been included in the training set has had more than 75% success. It can be observed that in some situations, the system may mistake what people are doing and cause classification errors, such as in the cafeteria environment; it can be seen that the sensors detected similarities in the movement and noise of people with the environments indoor soccer and conference room. This is probably because there are samples in the cafeteria with very quiet people (as in the conference) and samples in which there is high movement and many people (as in indoor soccer). In addition, the background noise data also vary greatly in all three scenarios. We assume the sensory system must be improved to include more information from people, such as recognition of facial expressions; these shortcomings will be reduced.

## 4. Discussion

The system allows one to properly label different types of rooms based on the detection of the actions people are doing. The assumption of being able to improve semantic labeling mechanisms for locations, based on what people do at these locations, has been confirmed. The results improve as more characteristics are taken into account. As future work, the sensory system must be improved. These improvements may include adding a Hokuyo laser to detect people with the leg detection algorithm, a face detection algorithm and better microphones. This will add new attributes to consider, such as the space arrangement of people talking or the facial expressions of the individuals in the environment.

This work shows the potential capacity of employing trained classifiers with unused features until now, and it proposes a labeling system. It must be considered that depending on the time of day, the circumstances can change. However, this system is initially intended to complement and improve other semantic labeling systems based on stationary elements. If used independently, it should be taken into account that the label the robot assigns to a room will be dynamic and will vary depending on what people do at that time. This dynamic feature is considered positive in order to offer an alternative to other existing labeling methods. Anyway, it can be stated that the information obtained through this method is useful.

As future work, it would be interesting to test and compare other learning methods described in the state of the art, such as neural networks.

## Figures and Tables

**Table 1:** Data obtained to differentiate library and cafeteria.

	Detected Label		
True Label	Library	Cafeteria	Total
Library	35 (97.22%)	1 (2.77%)	36
Cafeteria	1 (3.7%)	26 (96.29%)	27

**Table 2:** Results considering the twelve classifiers generated to differentiate library and cafeteria.

	Detected Label		
True Label	Library	Cafeteria	Total
Library	315 (98.43%)	5 (1.56%)	320
Cafeteria	2 (0.69%)	286 (99.3%)	288

**Table 3:** Data obtained using a good classifier to differentiate library and corridor.

	Detected Label		
True Label	Library	Corridor	Total
Library	22 (95.65%)	1 (4.35%)	23
Corridor	4 (12.9%)	27 (87.1%)	31

**Table 4:** Results considering several good classifiers.

	Detected Label		
True Label	Library	Corridor	Total
Library	51 (96.23%)	2 (3.77%)	53
Corridor	7 (13.2%)	46 (86.8%)	53

**Table 5:** Results obtained considering the experiment’s twelve classifiers.

	Detected Label		
True Label	Library	Corridor	Total
Library	305 (96.21%)	12 (3.78%)	317
Corridor	72 (22.15%)	253 (77.85%)	325

**Table 6:** Data obtained using a good classifier to differentiate library, cafeteria and corridor.

	Detected Label			
True Label	Library	Cafeteria	Corridor	Total
Library	31 (96.87%)	0 (0%)	1 (3.12%)	32
Cafeteria	0 (0%)	33 (97.06%)	1 (2.94%)	34
Corridor	2 (10%)	0 (0%)	18 (90%)	20

**Table 7:** Results obtained using several good classifiers to differentiate library, cafeteria and corridor.

	Detected Label			
True Label	Library	Cafeteria	Corridor	Total
Library	103 (92.79%)	3 (2.7%)	5 (4.5%)	111
Cafeteria	0 (0%)	133 (99.25%)	1 (0.75%)	134
Corridor	13 (13%)	0 (0%)	87 (87%)	100

**Table 8:** Results obtained with the sum of all twelve classifiers generated to differentiate library, cafeteria and corridor.

	Detected Label			
True Label	Library	Cafeteria	Corridor	Total
Library	301 **(93.19%)**	9 (2.78%)	13 (4.02%)	323
Cafeteria	1 (0.26%)	378 **(99.21%)**	2 (0.52%)	381
Corridor	60 (18.99%)	0 (0%)	256 **(81.01%)**	316

**Table 9:** Results obtained with the sum of ten classifiers to differentiate between corridor and exposition room, without background noise data.

	Detected Label		
True Label	Expo	Corridor	Total
Expo	207 (75.8%)	66 (24.2%)	273
Corridor	86 (29.1%)	209 (70.8%)	295

**Table 10:** Results obtained with the sum of ten classifiers to differentiate between corridor and exposition room, without people movement data.

	Detected Label		
True Label	Expo	Corridor	Total
Expo	156 (48.3%)	167 (51.7%)	323
Corridor	42 (16.2%)	217 (83.7%)	259

**Table 11:** Results obtained with the sum of ten classifiers to differentiate between corridor and exposition room, with complete data.

	Detected Label		
True Label	Expo	Corridor	Total
Expo	243 (80.2%)	60 (19.8%)	303
Corridor	64 (23.2%)	211 (76.8%)	275

**Table 12:** Results obtained using several good classifiers to differentiate library, cafeteria and corridor.

	Detected Label						
True Label	Exhibition	Indoor Soccer	Conference	Library	Cafeteria	Corridor	Total
Exhibition	269 (92.4%)	0 (0%)	0 (0%)	20 (6.8%)	0 (0%)	2 (0.6%)	291
Indoor soccer	0 (0%)	288 (92.6%)	11 (3.5%)	7 (2.1%)	19 (5.7%)	6 (1.8%)	331
Conference	8 (3.5%)	13 (5.7%)	164 (72.2%)	10 (4.4%)	17 (7.5%)	15 (6.6%)	227
Library	17 (5.4%)	2 (0.6%)	0 (0%)	289 (91.7%)	1 (0.3%)	6 (1.9%)	315
Cafeteria	1 (0.3%)	43 (14.8%)	47 (16%)	0 (0%)	202 (69%)	0 (0%)	293
Corridor	3 (1.2%)	1 (0.4%)	0 (0%)	42 (17.7%)	0 (0%)	191 (80.6%)	237

**Table 13:** Results obtained to test a new corridor scene with old data.

	Detected Label		
True Label	Corridor	No Corridor	Total
Corridor	70 (75.26%)	23 (24.73%)	93

## Abbreviations

The following abbreviations are used in this manuscript:

ROS	Robot Operating System
SVM	Support Vector Machine
